# From Competence by Time to Competence by Design: Lessons From A National Transformation Initiative

**DOI:** 10.5334/pme.1342

**Published:** 2024-03-25

**Authors:** Jason R. Frank, Andrew K. Hall, Anna Oswald, J. Damon Dagnone, Paul L. P. Brand, Richard Reznick

**Affiliations:** 1 Centre for Innovation in Medical Education, and Professor, Department of Emergency Medicine, Faculty of Medicine, University of Ottawa, Ottawa, ON, Canada; 2 Department of Emergency Medicine, University of Ottawa, Ottawa, ON, Canada; 3 Royal College of Physicians and Surgeons of Canada, Ottawa, ON, Canada; 4 Competency Based Medical Education, and Professor, Division of Rheumatology, Department of Medicine, Faculty of Medicine and Dentistry, University of Alberta, Edmonton, AB, Canada; 5 Department of Emergency Medicine, Queen’s University, Kingston, ON, Canada; 6 Standards and Accreditation, Royal College of Physicians & Surgeons of Canada, Ottawa, ON, Canada; 7 Clinical Medical Education, University Medical Centre and University of Groningen, the Netherlands; 8 Medical Education and Faculty Development, Isala Hospital, Zwolle, The Netherlands; 9 Queen’s University, Immediate Past President Royal College of Physicians and Surgeons of Canada, Canada

Competency based medical education (CBME) is a global movement to reform health professions education (HPE). In contrast to the traditional time-based training, it is an educational design approach that emphasizes necessary learner abilities, greater learner-centeredness, and better alignment with public’s needs for healthcare providers. Unlike numerous previous HPE innovations, CBME is framed as a complex transformational change with multiple proposed elements [1]. Training programs employing a competency-based approach can have five core components to operationalize their implementation with fidelity: outcome competencies, progressive sequencing, tailored learning experiences, competency-focused instruction, and programmatic assessment [2]. Health professions education institutions, disciplines, and programs across the globe have been implementing CBME at an ever-increasing rate. The CBME movement began with early innovations such as the CanMEDS framework [3,4,5,6,7], the ACGME Outcomes Project [8,9,10], entrustable professional activities [11,12], the Dutch CBME curriculum [13,14], the Cincinnati observable practice activities [15], the College of Family Physicians of Canada Triple C Curriculum [15,16,17], and the University of Toronto Orthopedic program [18]. CBME has now spread worldwide with implementations in, for example, Switzerland, Taiwan, Australian Orthopedic training [19], Finland, South Africa, Brazil, and many more countries. Competency based education is now a part of many health professions beyond medicine, including nursing [20] and veterinary medicine [21]. Despite the global implementation effort, the current discourse around CBME is still largely focused on theoretical underpinnings and potential [22,23] and countered with skeptical criticisms [24,25]. There are early efforts to evaluate and clarify the outcomes of these CBME curricula [26,27]. However, what is needed at this stage of the diffusion of these innovations is sharing CBME *praxis*: examples of the application of the CBME approach to real-life HPE systems and the lessons learned for others who follow.

In this special issue of *Perspectives on Medical Education*, a team of educators has come together to describe a unique Canadian configuration of CBME implementation called Competence by Design (CBD). Worldwide, the majority of CBME innovations have focused on implementing competency frameworks or programmatic assessment, two of the van Melle Core Components of CBME [28]. CBD was deliberately built using all of the five Core Components and best available design elements (such as competence committees) to address current challenges in medical training. CBD is the transformational change of specialty medicine education in Canada to a national, time-variable competency-based multi-specialty system for postgraduate medical education. The aim of this set of papers is to tell the story of CBD in the context of the greater global CBME implementation. These papers identify challenges and successes in CBME implementation at a national scale and articulate the unique approach taken by the Royal College of Physicians and Surgeons of Canada (Royal College) in the implementation of CBD, including the rationale, approach and methodology, tailored design elements, early outcomes and lessons learned. Note that these papers are not a series of how-to guidelines, nor are they framed as best practices. CBD is an example of CBME implemented in a real-life context, with successes and pitfalls. We hope that international readers will be able to see themselves and their programs in these descriptions and be accelerated on their unique path of large scale CBME implementation.

The CBD Special Collection is made up of nine papers:

In the flagship description of CBD, *Frank et al*. [29] describe the rationale for change and the key features and intentional design elements of CBD.

*Karpinski et al*. [30] then describe the details of the approach taken in implementing this national transformational change in postgraduate medical education.

To advance assessment *for* learning, *Richardson et al*. [31] elaborate on the approach taken to define coaching in the moment and coaching over time as part of CBD.

*Cheung et a*l. [32] describe the CBD approach to programmatic assessment using multiple assessment strategies over time and across contexts to support learner development and inform judgements about learner progress.

*Oswald et al*. [33] next discuss the nationally integrated strategy of Competence Committee design, faculty development and quality improvement, reflecting on the operationalization of key design features and lessons learned through challenges and evaluation.

*Bhanji et al*. [34] present perspectives on the role of high-stakes national assessment and examination in CBD and provide insight on how high-stakes examinations need to be adapted in competency-based national certification processes.

*Dalseg et al*. [35] describe the transition to an outcomes-based national accreditation system in parallel with CBD implementation, reflecting on accreditation as an enabler of CBME.

Describing key features of faculty and trainee development in CBD *Atkinson et al*. [36] highlight challenges, enablers, and lessons learned in facilitating the redesign of training programs for each specialty at the national level and supporting the implementation more generally across disciplines and programs.

Lastly, *Hall et al*. [37] describe the intentional incorporation of a robust national program evaluation strategy for CBD built around the three pillars of readiness to implement, fidelity and integrity of implementation, and outcomes, and share some early evaluation findings.

Like any transformational change process, the multi-year national launch of CBD has brought with it new challenges and unanticipated impacts. Six years of evidence from both formal program evaluation and feedback from partner groups provides clear signals that translating theory into practice is a complex task [38,39]. Further work is needed for any large-scale innovation like CBD to deliver on many of its initial promises of improved training. Readers from around the world will find in this collection a description of the efforts of medical educators to take the principles of CBME to the design and implementation of a major update to a functioning national system. Institutions and educators looking for examples of competency-based innovations, design elements, and pitfalls will find a rich selection in the nine papers describing the rollout of CBD.

## Disclaimer

The views and opinions expressed in this article are those of the authors and do not necessarily reflect the official policy or position of the Royal College of Physicians and Surgeons of Canada (“Royal College”). Information in this article about Competence by Design (“CBD”), its implementation and related policies and procedures do not necessarily reflect the current standards, policies and practices of the Royal College. Please refer to the Royal College website for current information.
